# Rapid Crop Cover Mapping for the Conterminous United States

**DOI:** 10.1038/s41598-018-26284-w

**Published:** 2018-06-05

**Authors:** Devendra Dahal, Bruce Wylie, Danny Howard

**Affiliations:** 1 Stinger Ghaffarian Technologies (SGT) Inc., Contractor to U.S. Geological Survey (USGS) Earth Resources Observation and Science (EROS) Center, Sioux Falls, SD USA; 2 USGS EROS Center, Sioux Falls, SD USA

## Abstract

Timely crop cover maps with sufficient resolution are important components to various environmental planning and research applications. Through the modification and use of a previously developed crop classification model (CCM), which was originally developed to generate historical annual crop cover maps, we hypothesized that such crop cover maps could be generated rapidly during the growing season. Through a process of incrementally removing weekly and monthly independent variables from the CCM and implementing a ‘two model mapping’ approach, we found it viable to generate conterminous United States-wide rapid crop cover maps at a resolution of 250 m for the current year by the month of September. In this approach, we divided the CCM model into one ‘crop type model’ to handle the classification of nine specific crops and a second, binary model to classify the presence or absence of ‘other’ crops. Under the two model mapping approach, the training errors were 0.8% and 1.5% for the crop type and binary model, respectively, while test errors were 5.5% and 6.4%, respectively. With spatial mapping accuracies for annual maps reaching upwards of 70%, this approach demonstrated a strong potential for generating rapid crop cover maps by the 1^st^ of September.

## Introduction

Spatially accurate and up-to-date land cover/land use (LCLU) datasets, including those with identifiable crop types, have been an essential source of information for various environmental modelling, monitoring, planning and research applications^1–4^. Crop cover maps have been used to study the relationship of agriculture with a range of factors such as environment, climate, socio-economy, human health and energy^5–11^. However, the importance and relevance of these crop cover maps depend on both a consistent quality in the historical time series and the latency of ongoing map production. Numerous studies and agencies have developed crop cover maps over a variety of spatial coverage with various temporal and spatial resolutions and crop classes^1–4,12^. For example, the National Agricultural Statistics Service (NASS) of the U.S. Department of Agriculture (USDA) has published the cropland data layer (CDL) annually since 1997 with 30 m or 56 m spatial resolution. Since the 2008 release, the annual NASS CDLs have been produced for the entire conterminous United States (CONUS) with prior releases including only a few selected states. However, these layers are not released before February of the following year due to processing constraints and other factors^3,13,14^. Friesz *et al*.^1^ modelled CONUS crop cover at a resolution of 250 m that included nine crops classes (corn, soybeans, sorghum, cotton, spring wheat, winter wheat, alfalfa, other hay/non alfalfa, and fallow/idle cropland) and all other crop types as ‘other’ crops at 250 m spatial resolution for 2000–2013 using the CDL as the model-dependent variable. Xiong *et al*.^15^ automated cropland mapping in Africa using a cloud computing technique, but generated only historical maps for 2003–2014 and did not focus on current years. Zhong *et al*.^14^ developed a method for rapid crop cover mapping, but included only two crops (corn and soybean) and was exclusive to the Corn Belt. Sakamoto *et al*.^16^ developed an algorithm and methodology for mapping crop cover in near real time and predicting yields, but like Zhong *et al*.^14^, they focused only on corn and soybeans.

As a result of recent advancements in rule-based decision tree modelling, Geographic Information Systems (GIS), remote sensing, computer technologies, and data mining approaches are being leveraged for the rapid mapping of local to global LCLU datasets^1,2,4,15,17–19^. Despite the extensive improvements, little emphasis has been given to generating LCLU maps for large areas, such as the CONUS, in a real or near real-time production environment.

In this study, we tested advanced data mining technologies to develop CONUS-wide rapid crop cover maps with 250 m resolution that included the following classes: 1) corn, 2) soybeans, 3) sorghum, 4) cotton, 5) spring wheat, 6) winter wheat, 7) alfalfa, 8) other hay/non alfalfa, 9) fallow/idle cropland and 10) other crops. The purpose was to identify the earliest viable month of the year for production of rapid annual crop cover maps with minimized test and training errors and maximized spatial mapping accuracy of the annual crop maps, tested by random sampling.

A secondary objective of this study was to address/reduce the error of commission in classifying ‘Other crops’ which was observed by Friesz *et al*.^1^. We hypothesized that crop cover maps can be produced by the beginning of the major crop harvesting periods while remaining within 0.5%, 1.5% and 5%), respectively, of the pure pixel training, the pure pixel test (excluded the years 2014–2016), and ‘spatial mapping’ accuracy (500,000 random mixed and pure pixels per year across all years, of Friesz *et al*.^1^ (hereafter referred to as the ‘baseline study’).

## Methods

### Study Area

The study area includes the agricultural areas of the CONUS, which extends within the boundaries of 24.5 N to 49.5 N latitude and 66.95 W to 124.76 W longitude. This area is based on the Cultivated crops (Class 82) and Pasture/hay (Class 81) classes from National Land Cover Database (NLCD)^17,20,21^.

### Input datasets and modelling

This study utilized the same input datasets (Table 1) and modelling software (RuleQuest See5) as described in the baseline study. However, the methodology was modified in accordance with determining the earliest time of year at which crop cover could be accurately classified, as per comparison with the model training data (NASS CDL).

**Table 1:** Data layers used as independent variables for development of the Crop Classification Model (CCM) and to generate spatial maps.

Type	Name	Acronym	Temporal Resolution	Date Range	Remarks
Normalized Difference Vegetation Index (NDVI)	Smoothed eMODIS Terra Collect 5 NDVI	SMNDVI	Weekly (52)	2008–2016	1
Phenology	Amplitude	AMP	Annual	2008–2016	2
	Duration	DUR	Annual	2008–2016	2
	End of Season NDVI	EOSN	Annual	2008–2016	2
	End of Season Time	EOST	Annual	2008–2016	2
	Maximum NDVI	MAXN	Annual	2008–2016	2
	Time of Maximum NDVI	MAXT	Annual	2008–2016	2
	Start of Season NDVI	SOSN	Annual	2008–2016	2
	Start of Season Time	SOST	Annual	2008–2016	2
	Time Integrated NDVI	TIN	Annual	2008–2016	2
Weather	Precipitation	PPT	Monthly (12)	2008–2016	3
	Maximum Temperature	TMAX	Monthly (12)	2008–2016	3
	Minimum Temperature	TMIN	Monthly (12)	2008–2016	3
	Mean Temperature	TMEAN	Monthly (12)	2008–2016	3
Climate	30-Year Precipitation Normal	C_PPT	Static	N/A	
	30-Year Maximum Temperature	C_TMAX	Static	N/A	
	30-Year Minimum Temperature	C_TMIN	Static	N/A	
	30-Year Mean Temperature	C_TMEAN	Static	N/A	
Geophysical	Major Land Resource Area	MLRA	Static	N/A	
	Digital Elevation Model	DEM	Static	N/A	
	Aspect	ASP	Static	N/A	
	Slope	SLP	Static	N/A	
	Irrigation	IRR	Static	N/A	
	SSURGO Soil Organic Carbon	SOC	Static	N/A	
	SSURGO Available Water Capacity	AWC	Static	N/A	
	SSURGO Bulk Density	BD	Static	N/A	
	SSURGO Clay Content	CLAY	Static	N/A	
	Omernik Ecoregion Level III	ECO	Static	N/A	

Note: CCM model was developed with temporal variables only for 2008–2013 but utilized all static variables. Numbers (1, 2, and 3) in the Remarks column refer to the following: 1 - weekly layers after 35 were taken out of final model, 2 - all of these layers were taken out of the final model, and 3 - all months after August were taken out of the final model.

The Normalized Difference Vegetation Index (NDVI) computed from multispectral satellite data has been widely used for many years to measure and monitor vegetation growth, cover and biomass^2,4,22^. The U.S. Geological Survey (USGS) Earth Resources Observation and Science (EROS) Center has been generating and distributing Moderate Resolution Imaging Spectroradiometer (MODIS) based NDVI composites with 250 m resolution called eMODIS^23^. We acquired eMODIS Terra Collect 5 weekly composites for 2008–2013 and Aqua Collect 6 weekly composites for 2014–2016 for the CONUS. The raw eMODIS data generally contains noisy pixels introduced by clouds, aerosols as well as changing illumination patterns. Therefore, these raw weekly composites were temporally smoothed using a weighted, least-squares linear regression approach, which involves a moving temporal window of ±5 composites to calculate a regression line. The window is moved one period at a time, resulting in a family of regression lines associated with each data point. This family of lines is then averaged at each point, and interpolated between points, to provide a continuous, relatively smooth NDVI signal over time. Furthermore, since the phenomena that introduce noise into raw satellite data usually reduce NDVI values, a weighting factor was applied during the smoothing process that favors peak points over slope or valley points. A final operation assures that all peak NDVI values in the moving window are retained.

Remote sensing phenology datasets that identify and measure nine different phenological metrics of vegetation were acquired for 2008–2016 from https://phenology.cr.usgs.gov. These metrics were based on eMODIS and identified as start-of-season time (SOST), start-of-season NDVI (SOSN), end-of-season time (EOST), end-of-season NDVI (EOSN), maximum NDVI (MAXN), maximum NDVI time (MAXT), duration of season (DUR), amplitude of NDVI (AMP), and time-integrated NDVI (TIN).

Datasets related to weather and climate, such as monthly and longterm average precipitation (PPT), maximum temperature (TMAX), minimum temperature (TMIN), and mean temperature (TMIN) were downloaded from the PRISM Climate Group at http://prism.oregonstate.edu/.

The NASS CDL data for years 2008–2016 were obtained from the NASS CropScape application (https://nassgeodata.gmu.edu/CropScape/). The annual CDL datasets, which come in either 30 m or 56 m spatial resolutions, were resampled to 250 m using a majority resampling method. To simplify the modelling process, crop classes of the CDL datasets were narrowed down from over 100 crops classified in the CDL, to the 9 most abundant crops in the CONUS (corn, soybeans, sorghum, cotton, spring wheat, winter wheat, alfalfa, other hay/non alfalfa, and fallow/idle cropland), with all other crop classes lumped as ‘Other’. The narrowed crop classes were masked out by NLCD agriculture class (explained in Study Area section) to make sure they were within the defined study area. Six of the nine years (2008–2013) of resampled 250 m CDL datasets were used in the training of the rapid Crop Classification Model (CCM), with the remaining 3 years (2014–2016) used only for map validation purposes.

Prior to model training, a filtration procedure was followed to systematically select the specific pixels that had the highest probability of containing only one single crop type, which would qualify it for use in the model training process. A 250 m pixel only qualified for model training if it was 1) entirely surrounded by other pixels of the same crop type in a moving 3 × 3 pixel window and 2) 100 percent contained by an unbroken patch of the pre-sampled CDL layers (30 m or 56 m depending on the year of the data) – referred to as ‘pure pixels’. Through this filtration process, the specific phenological characteristics of each crop type were brought into focus^24^. The selected 250 m pixels from each annual resampled 250 m CDLs were converted to point features and mosaicked to create one training dataset. One drawback to this process was an oversampling of the ‘Other’ crops class by a factor of 10, relative to any of the known crop types, which would introduce a modelling bias^24,25^. To address this bias, a more equal representation was obtained by randomly removing all but 10% of the samples associated to the ‘Other’ class. The final training pixel locations were used to extract values from the list of independent variables (Table 1), as defined by the baseline study and Howard and Wylie^2^.

The extracted records were compiled to create the model sample database, which included 12,765,948 records. The model sample database was randomly divided into two sets made up of 90 and 10% of the total records. The 90% database was used for training and development of the model (model training database), whereas the 10% database was withheld from training and explicitly used to test model performance (model pure pixel test database). Due to the concern of possible duplication between training and test dataset, as well as concern of under representation of rare crop type, no repeat random-sampling was implemented. The 90 and 10% sampling approach was a continuation of the baseline study and Howard and Wylie^2^. It is important to note that this dataset excluded the years 2014–2016.

In this study, a decision tree classifier, RuleQuest See5 software (version 2.07 GPL - https://www.rulequest.com/see5-info.html), was used to develop the classifier models. See5 has been used extensively for data mining, delineating categories, and making predictions based on training data records consisting of a dependent variable and a series of independent variables^2,26,27^. It includes robust methods, such as adaptive boosting, an ensemble method that has demonstrated to enhance classification accuracy and to reduce noise sensitivity. See5 is regarded as well-established algorithm among machine learning community and highly suited for classification of remote sensing data as it is robust and perform well with large datasets in a time efficient way^26,28–31^. The algorithm is generated based on a set of if-then rules and is much simpler to understand; however, it has tendency to over-fit if not paid attention to training and test accuracy difference. This overfitting can be corrected by bringing these accuracies closer.

In an effort to identify the ultimate rapid mapping capability of the CCM, multiple modelling iterations were conducted always applying 5 boosting trails and allowing a winnow option but incrementally removing weekly and monthly input data from the model training. The subsequent model training, test, and ‘spatial mapping’ accuracies were evaluated to identify the point of maximum training and test accuracies in relation to the temporal cut-off date (day of year) and the original CCM. The training and pure pixel test accuracies are based on the model samples and represent the accuracy of model classification rules prior to 2014. The ‘spatial mapping’ accuracy is based on a random sample of all the mapped crop type pixels through time (2008–2016) and space. The ‘spatial mapping’ accuracy was derived from the comparison between modelled crop cover pixels (not limited to pure pixels) and applicable NASS CDL classifications at mixed and pure pixels.

Once the earliest viable processing date with acceptable training and test accuracies and ‘spatial mapping’ accuracies was established, the focus was shifted to normalizing the user’s and producer’s accuracies and reducing the overestimation of the ‘Other’ crop class, which were issues observed in the baseline study. An improperly proportioned sample database can cause decision tree algorithms such as See5, to have a bias towards the largest set of homogeneous sample classes^25^. This can lead to a high rate of over-fitting commission errors in the large classes and omission errors in the relatively smaller classes. We believed this disproportion of sample records was partly the reason why the original CCM in the baseline study was overestimating the ‘Other’ crop class. In addition, if the class represents a vast mix of crop types, it tends to force the decision tree rules to not only be biased towards ‘Other’ crops based on its high frequency of occurrence, but also the decision tree rules can be very broad and inclusive in an attempt to capture the diversity of crops that fall within ‘Other’ crops. We hypothesized that all of these factors lead the original CCM model of the baseline study to over-classify ‘Other’ crops, at the expense of the other specific crop classes.

**Figure 1:**
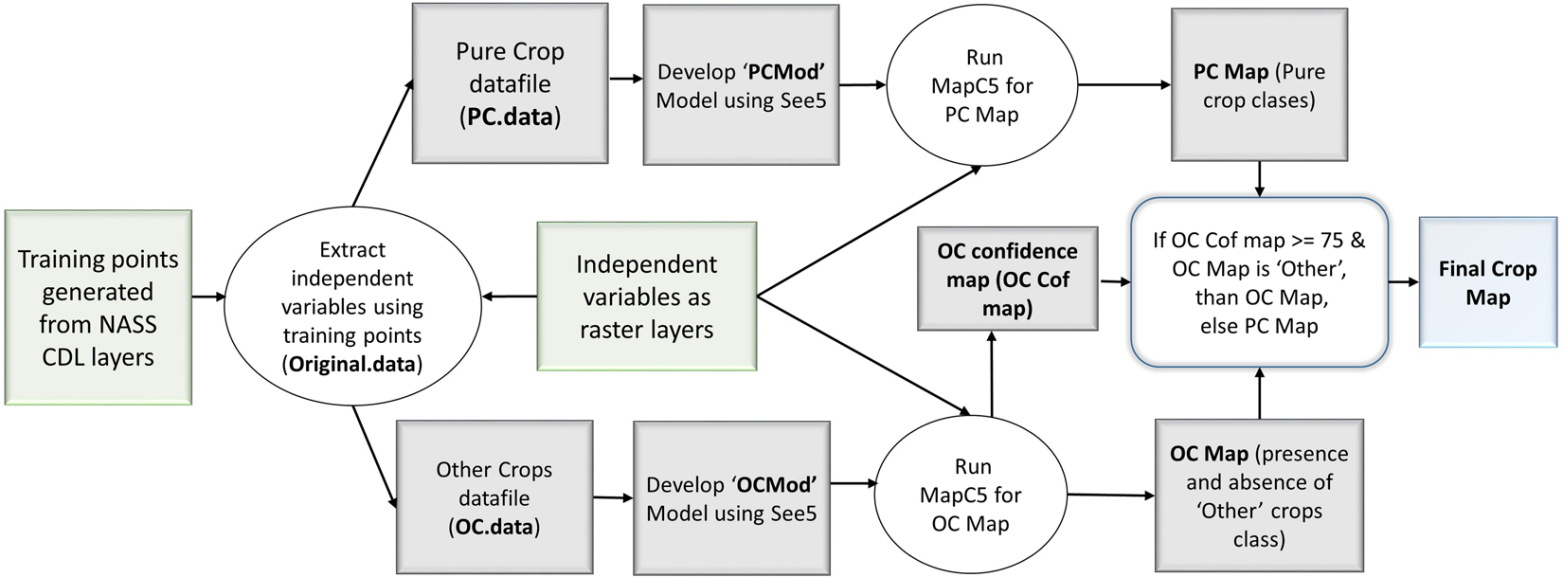
Flowchart explaining two model mapping approach to generate final crop cover map. Original datafile contains Corn, Cotton, Sorghum, Soybeans, Spring wheat, Winter wheat, Alfalfa, Other hay/Non alfalfa, Fallow/Idle cropland and ‘Other’ crop classes; PC datafile contains Corn, Cotton, Sorghum, Soybeans, Spring wheat, Winter wheat, Alfalfa, Other hay/Non alfalfa, Fallow/Idle cropland; and OC datafile is presence and absence of ‘Other’ crops and Final Map contains all classes listed in original datafile.

To address this issue, we implemented a two model mapping approach that separated the ‘Other’ class from the specific crop classes (corn, soybeans, sorghum, cotton, spring wheat, winter wheat, alfalfa, other hay/non alfalfa, and fallow/idle cropland). This separation was accomplished by developing two decision tree models; one for the specific crop classes, the Pure Crop Model (PCMod) and a second, the Other Crop Model (OCMod), a binary decision tree model to classify the presence or absence of the ‘Other’ class (see Fig. 1). Both of these models utilized the same input datasets (independent variables as listed in Table 1) and model parameters; however, usage of the variables varied by the models (See Supplementary Outfile S1 for PCMod and Outfile S2 for OCMod, respectively).

### Mapping crop covers

Following the development of the two mapping model approach, MapC5 was used to spatially implement the models to generate maps for 2008–2016 for the entire study area. MapC5, developed by the USGS EROS Center, is an application based on publicly available source code provided by RuleQuest (http://www.rulequest.com) to parse the decision-tree model files and apply them to specific input cases. This code was integrated with an open source raster input/output library (GDAL: http://www.gdal.org) to produce applications that read a list of raster images corresponding to the independent variables in the decision tree model on a pixel by pixel basis, apply the model classification rules and sub-rules, and write the resulted class to the corresponding pixel to a new output raster image. In addition to the classification map, the MapC5 software also generates a confidence map with pixel values that represent the percent of the training observations, at each respective prediction rule set, that were correctly classified. Using the confidence maps that were derived during the implementation of the OCMod, a percent probability map of the ‘Other’ crops was created and used for merging the classification results from the OCMod and PCMod. Classification results from the OCMod were given preference over that of the PCMod when the percent probability map of the ‘Other’ crops was greater than or equal to 75% and classified as ‘Other’. All other pixels for the final crop maps were from the classification results of the PCMod. Through this process, the output crop cover maps from the PCMod and the OCMod were merged to generate final rapid crop cover maps for 2008–2016. Figure 1 shows an illustration of the two model mapping approach to generate final rapid crop cover maps.

### Accuracy Assessment

To assess the ‘spatial mapping’ accuracy of the classified rapid crop cover maps, a comparison was made between the modelled results and the NASS CDLs. For this comparison, a set of 500,000 points was randomly sampled across time and space and used to extract pixel values from both crop cover products for use in a statistical analysis. The resulting information was formatted into a confusion matrix that revealed the producer’s and user’s accuracies for individual crop type and overall ‘spatial mapping’ accuracy by applying the equations below:

1 $${\rm{Overall}}\,{\rm{accuracy}}=\frac{\,sum\,of\,correctly\,classified\,pixels\,for\,all\,crop\,types}{total\,number\,of\,pixels\,for\,all\,crop\,types}\times 100\,$$

2 $$\text{Producer}\mbox{'}{\rm{s}}\,{\rm{accuracy}}=\frac{number\,of\,correctly\,classified\,pixels\,of\,a\,crop\,type}{total\,number\,of\,the\,crop\,type\,pixels\,in\,the\,CDL\,map}\times 100$$

3 $$\text{User}\mbox{'}{\rm{s}}\,{\rm{accuracy}}=\frac{number\,of\,correctly\,classified\,pixels\,of\,a\,crop\,type}{total\,number\,of\,the\,crop\,type\,pixels\,in\,the\,classifed\,map}\times 100$$

The producer’s accuracy was calculated for each cover type in the NASS CDL as reference and indicates the probability that a NASS CDL pixel was correctly mapped (across all crop types) and measures errors of omission. An omission error occurs when a pixel is excluded from the category to which it belongs in the validation dataset. The user’s accuracy indicates the probability that a pixel from the rapid crop cover map matches the NASS CDL and measures errors of commission. The commission error occurs when a pixel is mapped in an incorrect category relative to the validation data. For classification mapping accuracy assessments, such as this, it is extremely important to take into consideration errors of omission and commission, in supplement to the user/producer accuracies. Overall accuracy indicates what proportion of the NASS CDL pixels were mapped by the rapid crop cover map correctly. The overall accuracy is calculated as a percent, with 100% accuracy being a perfect classification where all reference pixels were classified correctly^32^.

## Results

A number of tests were conducted to assess the feasibility of a rapid application of the crop cover classification model. Each iteration yielded a differing level of model error and mapping agreement in the NASS CDL comparison test. To gain further context, iterative rapid mapping scenario model error and mapping results were compared to that of the baseline study, which utilized the full set of multi-temporal data. All of these iterations were performed in an attempt to identify a rapid mapping model permutation that did not compromise mapping accuracy more than 5% from the baseline study and showed little or no overfitting tendencies^33^.

**Figure 2:**
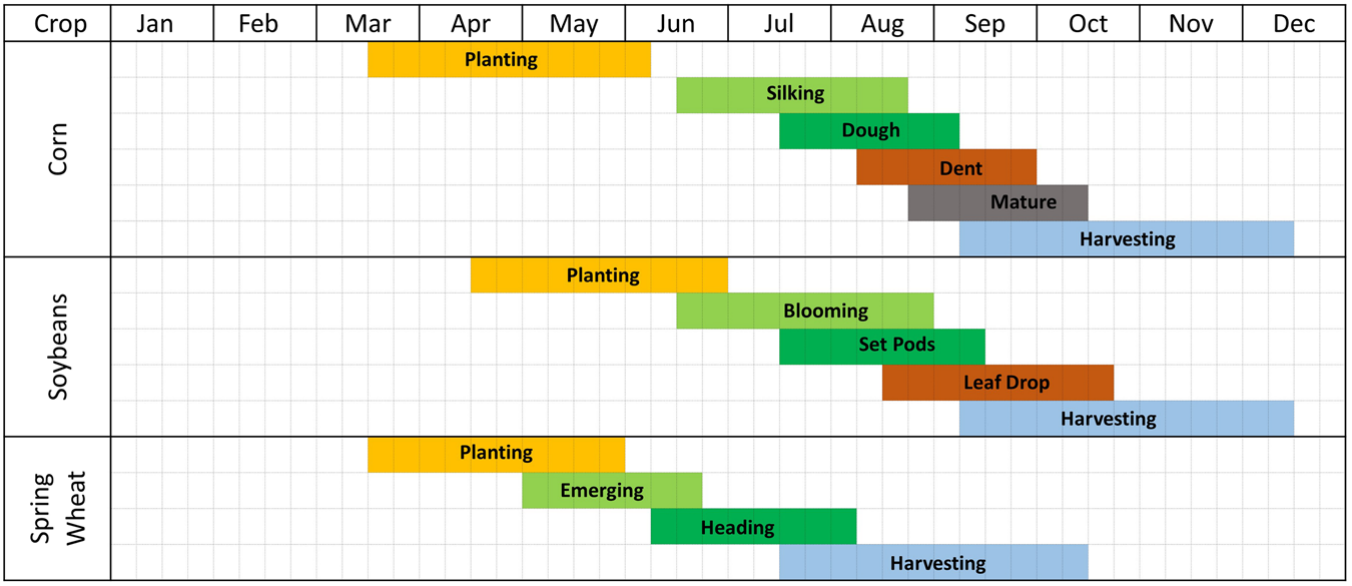
Example of general crop calendar for three crop types in CONUS. Note: the stages are overlapped because crop progress vary by latitude.

Most of the rapid mapping scenario modelling iterations produced error and accuracy statistics that were comparable to that of the baseline study. Table 2 shows that there was a possibility of producing a crop cover map as soon as the 1^st^ of July (see column ‘July’), but it would require the availability of all the annual and static input data layers. Unfortunately, because a full set of the eMODIS^23^-based annual phenology metrics suite is not typically available until after July of the following year (https://phenology.cr.usgs.gov/index.php), this suite of metrics was not useful to the rapid mapping application. However, excluding the phenology metrics suite and executing the rapid mapping on the 1^st^ of September (version SepNP) would produce acceptable results within 1.5% of baseline annual overall accuracies (Table 2). This finding supports the feasibility of rapid crop mapping approximately coinciding with the start of major harvesting efforts. All crops have a different calendar of planting, maturing, and harvesting and these varies by the latitude and weather condition. Figure 2 shows an example of a generic national calendar for three major crops in U.S.; corn, soybeans, and spring wheat^34^.

**Table 2:** Summary results from selected pure pixels test iterations (upper panel) and overall spatial accuracy for each mapping year (lower panel) in comparison with the baseline^1^.

	Baseline	Oct	Sep	Aug	July	June	SepNP	AugNP	SepNP2m	
Training accuracy (%)	99	98.9	98.9	98.7	98.7	98.5	**98.6**	98.1	99.2*	**98.5^**
Test accuracy (%)	92.4	92.4	92.2	91.6	91.1	90.4	**91.7**	89.9	94.5*	**93.6^**
**Overall spatial accuracy of modelled maps from 500 k sampled points**.										
Year										
2008	74.31	74.12	73.93	73.6	73.26	58.11	**73.35**	71.02	**69.88**	
2009	65.39	65.32	65.2	65.04	65.04	58.18	**64.98**	63.29	**64.28**	
2010	67.78	67.86	67.69	67.44	67.28	55.05	**67.54**	64.62	**67.19**	
2011	66.74	66.71	66.61	66.35	65.93	59.59	**66.39**	63.64	**66.31**	
2012	67.65	67.63	67.53	67.28	67.07	53.82	**67.31**	63.2	**67.09**	
2013	66.81	66.88	66.73	66.36	66.2	65.89	**65.54**	63.03	**64.31**	
2014									**59.62**	
2015									**57.57**	
2016									**58.03**	

(All processing was as of the 1^st^ day of the month, NP is no phenology, 2 m is two model mapping approach, *the PCMod, ^the OCMod, bold text indicates best results with all timely available input variables).

Version SepNP exhibited continuity with the baseline study but excluded all annual phenology metrics and monthly and weekly variables for time intervals after the 1^st^ of September. The model’s training and pure pixel test accuracies for version SepNP were 98.6% and 91.7%, respectively and, the annual overall ‘spatial mapping’ accuracies (including mixed pixels) for the SepNP were between 64.98% and 73.35% for 2008–2013 (Table 2). The difference of model training and test accuracies between the baseline study and the SepNP were 0.4% and 0.7%, respectively and the average (2008–2013) overall ‘spatial mapping’ accuracy difference between the baseline study results and the SepNP results was only 0.6%.

**Figure 3:**
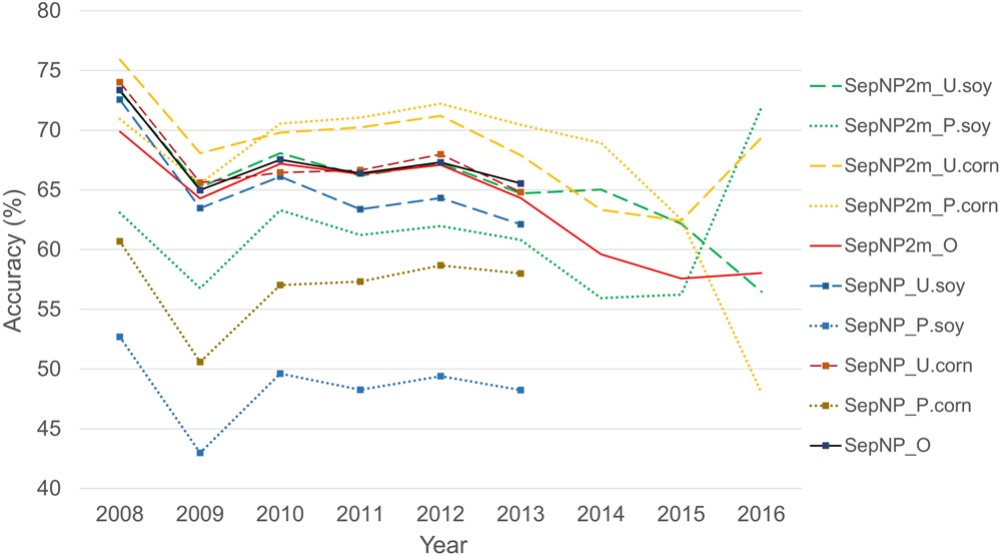
Comparison of spatial map accuracies (overall accuracy “O”, producer’s accuracy “P” and user’s accuracy “U”) from single model (SepNP) and two model mapping approach (SepNP2m) based on 500,000 random sample points which included mixed pixels for corn and soybean.

**Figure 4:**
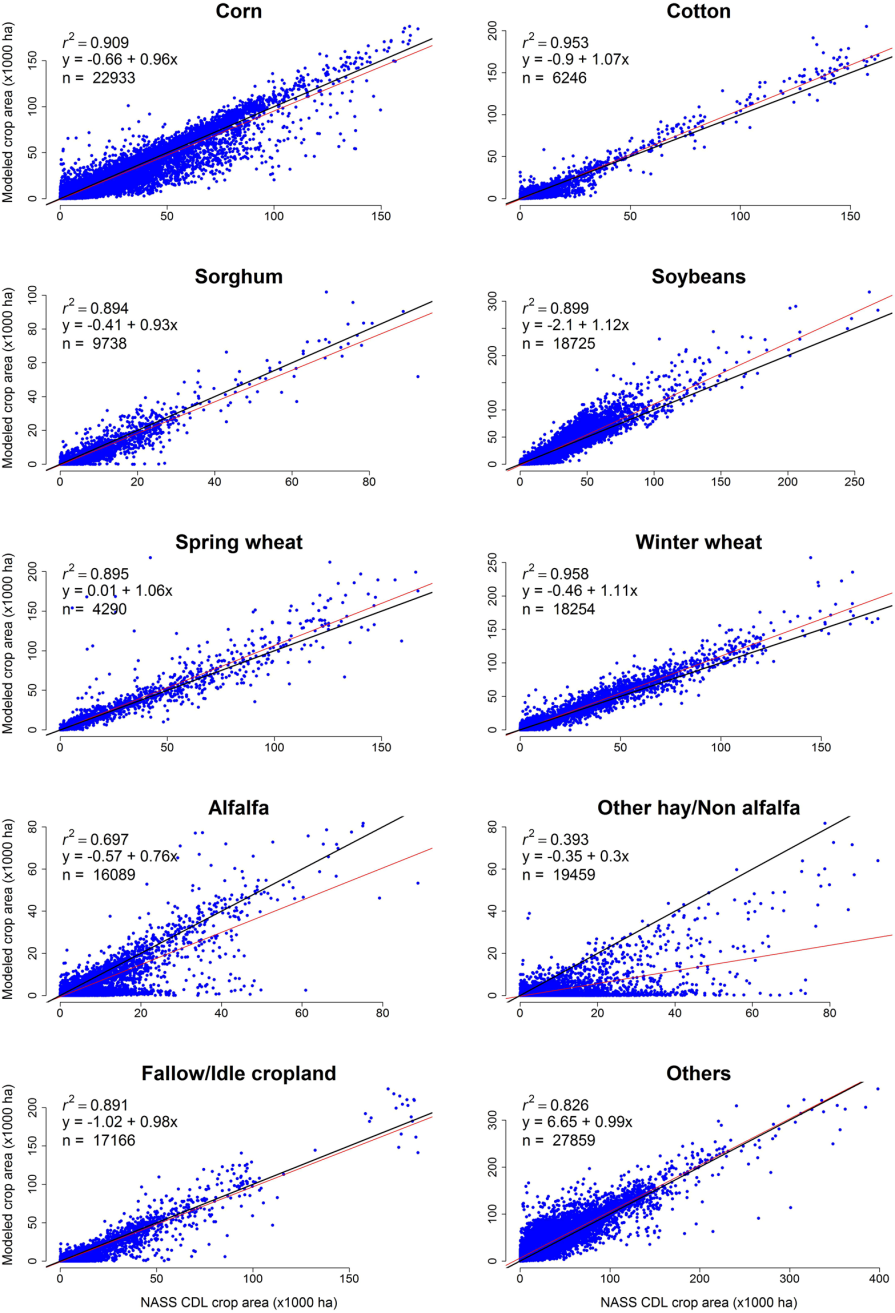
Scatterplots comparing total crop acreage (modelled against resampled NASS CDLs-250 m) of each crop types within CONUS counties for all mapping year (2008–2016). The black line is 1:1. The red line is linear fit. ‘n’ denotes total number of points (counties by years) included in the scatterplots.

**Figure 5:**
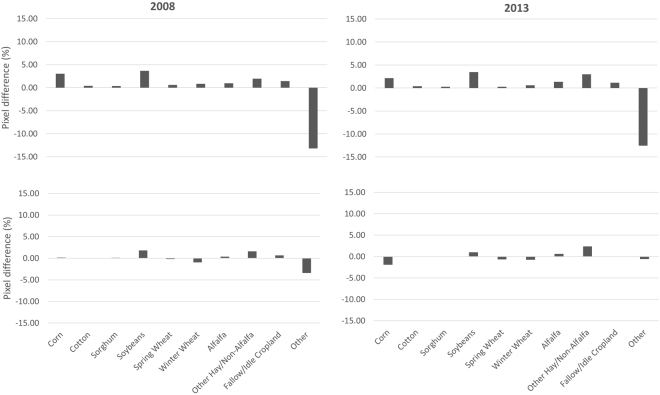
Comparison of pixel differences by crop types for maps of 2008 and 2013 between resampled NASS CDL 250 m and baseline study (upper panels) and SepNP2m (lower panels).

According to our initial objective, the results of the SepNP rapid crop cover mapping model were within the targeted acceptable range of the baseline study results. However, there were noticeable differences between the averaged user’s and producer’s accuracies (Fig. 3). The user’s accuracies were consistently higher than the producer’s accuracies, which indicates a significant percentage of omission error. A comparison of individual crop area between the original modelled maps from the baseline study and NASS CDL maps revealed that the major row crops such as corn, cotton, soybeans and winter wheat were mapped with higher producer’s and user’s accuracies, while alfalfa, other hay/non alfalfa and fallow/idle cropland had very low producer’s accuracies and higher omission errors (Table 3). Conversely ‘Other’ crops had high producer’s accuracies but low user accuracies, relatively high commission errors. Therefore, we developed and implemented the two model mapping approach (version SepNP2m) in an attempt to address this issue. The crop cover map results from the two model mapping approach were compared against the NASS CDL maps on a countywide acreage of all individual crop types, and the model was found to have performed well, mapping a similar amount of area and with high R^2^ values with only exceptions being alfalfa and other hay/non alfalfa crop types (Fig. 4). This was a significant improvement observed over the baseline study (Fig. 5). Through the two model mapping approach, the overestimation of the ‘Other’ crop class by the baseline study was substantially reduced. For example, the baseline study had estimated 13.21 and 12.55% more pixels for ‘Other’ crop for 2008 and 2013, respectively, when compared to the NASS CDL, whereas those differences were reduced to 7.32 and 2.39%, respectively, by the two model mapping approach. The differences of mapped areas for all of the individual crop types were improved by the two model mapping approach (Fig. 5). However, while the two model approach was found to significantly improve the producer’s accuracies, there was almost no change to the user’s and overall accuracies (Fig. 3). This finding was likely due to forcing the PCMod to make a crop type classifications on pixels formerly grouped in the frequent, heterogeneous, and overestimated catch-all category of ‘Other’.

**Table 3:** Comparison of user’s and producer’s accuracies for random (n = 500,000) pure and mixed pixels (spatial accuracy) from three models: Baseline^1^ (2008–2013) September no Phenology (SepNP; 2008–2013) and September no phenology with two model mapping approach (SepNP2m; 2008–2016).

	Year	Corn		Cotton		Sorghum		Soybeans		Spring Wheat		Winter Wheat		Alfalfa		Other Hay/Non Alfalfa		Fallow/idle cropland		Other Crops	
		U	P	U	P	U	P	U	P	U	P	U	P	U	P	U	P	U	P	U	P
Baseline	2008	76.07	62.25	78.28	62.98	69.09	44.29	75.56	54.97	72.83	56.52	74.20	64.44	69.50	36.84	79.24	12.85	64.12	29.05	74.00	93.24
	2009	66.81	50.74	74.20	56.06	58.50	33.93	64.69	44.00	66.01	45.36	71.74	57.06	63.83	30.00	76.79	12.27	63.95	42.75	64.45	90.83
	2010	66.78	57.57	73.66	59.92	61.45	41.25	66.67	49.58	65.23	51.32	70.48	64.10	63.65	28.51	70.29	7.23	64.88	47.69	68.17	89.18
	2011	67.65	57.50	73.49	63.00	55.38	35.81	64.34	48.95	64.78	52.06	68.61	61.30	62.20	25.67	70.25	10.95	63.49	43.41	66.85	88.86
	2012	68.45	59.22	74.25	62.78	60.91	43.02	64.95	49.80	60.85	55.20	69.25	66.15	63.00	27.95	69.11	11.02	63.17	44.98	68.16	88.15
	2013	66.46	59.27	73.19	60.09	56.48	44.77	64.72	49.64	61.75	54.46	68.64	62.34	61.76	29.96	64.71	14.39	62.46	43.96	67.70	87.48
SepNP	2008	74.01	60.69	77.84	62.93	66.85	43.34	72.57	52.69	71.99	56.1	73.24	64.44	68.15	36.01	76.66	12.99	62.82	29.33	73.65	92.67
	2009	65.61	50.58	73.22	55.63	56.29	33.48	63.47	42.98	66.48	46.32	71.32	56.54	62.49	30.03	77.46	12.28	63.78	42.31	64.31	90.62
	2010	66.46	57.03	73.3	59.88	60.36	40.06	66.11	49.62	65.69	51.11	69.68	64.11	64.17	27.85	71.76	7.24	64.98	46.70	68.01	89.08
	2011	66.66	57.33	73.17	62.19	54.39	35.49	63.39	48.26	64.5	52.11	68.02	60.97	62.78	24.84	69.54	11.50	63.09	42.63	66.81	88.63
	2012	67.97	58.68	74.45	63.00	57.72	40.95	64.33	49.39	60.93	54.17	68.74	65.33	63.15	27.95	70.13	10.99	63.1	45.01	67.89	88.10
	2013	64.8	57.99	70.3	58.05	50.87	39.82	62.13	48.25	59.56	52.75	67.42	61.1	60.54	28.9	62.48	11.43	59.22	40.77	67.08	86.78
SepNP2m	2008	75.92	70.93	76.68	73.11	63.58	52.32	73.26	63.09	68.03	65.76	71.02	73.02	64.56	48.80	57.67	22.12	61.48	41.81	65.36	81.00
	2009	68.08	65.54	73.06	69.18	57.30	46.57	65.23	56.77	62.24	59.73	72.66	68.49	60.09	44.62	69.09	23.06	61.69	56.69	59.49	75.83
	2010	69.79	70.54	73.72	73.07	63.17	53.50	68.07	63.31	62.99	64.28	71.71	74.01	59.97	42.18	60.37	15.55	64.01	60.90	64.38	73.08
	2011	70.23	71.06	75.35	77.02	55.01	45.88	66.18	61.22	62.86	65.36	69.51	71.83	59.74	39.63	63.20	21.54	62.63	56.59	62.61	72.55
	2012	71.21	72.21	74.68	75.83	63.20	53.63	67.23	61.98	58.36	68.05	70.17	76.15	59.76	41.67	61.69	21.79	62.05	59.01	63.88	70.67
	2013	67.91	70.44	72.12	70.75	54.60	50.21	64.71	60.82	56.71	66.92	68.04	70.50	57.39	41.78	57.27	23.55	57.95	55.09	62.11	67.93
	2014	63.32	68.93	66.59	62.34	47.42	40.79	65.02	55.94	55.65	59.04	67.08	61.66	58.83	33.53	39.23	6.08	55.10	50.60	52.08	66.80
	2015	62.42	62.52	55.68	60.38	51.77	32.64	62.16	56.23	54.86	61.42	61.27	69.52	48.35	37.47	24.79	5.31	59.27	35.47	51.05	65.40
	2016	69.39	47.99	63.49	60.13	45.59	39.74	56.47	71.94	50.42	60.28	61.87	74.54	52.96	35.08	28.54	6.83	62.27	39.52	52.46	64.98

U is user’s accuracy, P is producer’s accuracy.

**Figure 6:**
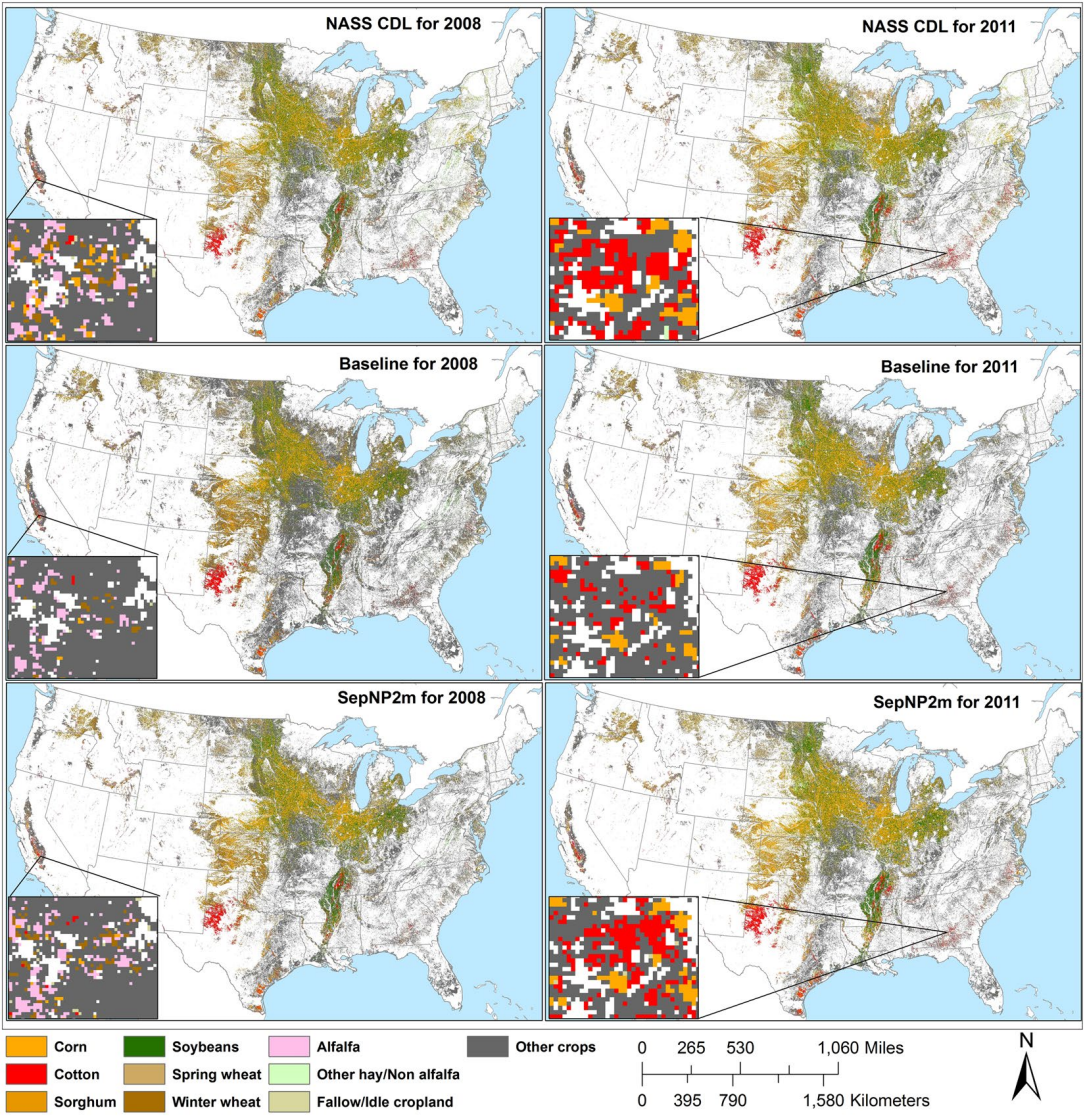
Visual comparison between NASS CDL 250 m, baseline study, and rapid map developed as of the 1^st^ of September by the two model mapping (SepNP2m) for 2008 and 2011. Insets are zoomed-in areas to highlight the differences. (Map generated in ArcGIS 10.3.1.).

Finally, the rapid crop cover maps with a 250 m spatial resolution were produced for 2008–2016 by merging the results of the two model mapping approach (see Supplementary Fig. S1). A simple visual assessment suggests that the rapid crop cover map products maintained overall spatial distribution and patterns of the crop cover that were observed in the resampled, 250 m NASS CDL. Some of the regional accuracy fallouts that were documented in the baseline study were minimized by the new mapping approach (Fig. 6). The two zoomed-in areas of Fig. 6, one in the Southeastern Coastal Plain, Georgia, and another in Central Valley, California, show examples of the fallout in the baseline study—subtle corrections made by the two model mapping approach are evident in the amounts of other crop shown. However, this current study was still unable to correct some regional error noted in the baseline study, such as the Pennsylvania-Maryland border in 2008 and Iowa-Missouri border in 2011 (Fig. 6).

## Discussion

We believe this study demonstrates that it is possible to accurately and rapidly map crop cover for the CONUS before harvesting begins for most of the major crops. These results could provide useful information to applications that need timely crop type estimates with a consistent synoptic history (e.g., possible near real time carbon flux estimates^35^, regional water usage^36–38^, or assessment of policy or economic impacts on crop rotations and extents^7,39^). Though the 250 m spatial resolution of the rapid crop cover maps falls short of datasets such as was used in our training (30/56 m NASS CDL), the 250 m resolution presented in this study holds sufficient ground resolution to study the dynamics of crop cover and crop-related land use^35,40^ for major crops that tend to have large field sizes. We found that the total area of individual crop types classified by the two model mapping approach closely matched that of the resampled 250 m NASS CDL, which carries high overall accuracies for the large-area row crops (upwards of 90%^3,41,42^). Although the overall area coverage for each crop is in close agreement with NASS CDL (Fig. 5), some concern remains with the producer’s and user’s accuracies for some crop classifications having clear omission and commission errors. Mapping inaccuracies such as these could potentially be remediated by further optimizing the training sample for each class, for example, normalizing sample proportions and defining the minimum and maximum number of training points for each class^43^.

In our testing, we observed that initiating mapping prior to September 1^st^ produced a less promising result, which agreed with the finding of Zhong *et al*.^14^, that remote sensing best captures and distinguishes crop phenology sometime after the crops reach peak growth stage (approximately mid-August in larger parts of the CONUS study area). Johnson^44^ also found August MODIS NDVI values hold optimal information when mapping corn and soybean yield in the U.S. Corn-Belt. Accordingly, we found August eMODIS NDVI and climate variables were the most important component for improving the map accuracy in our study.

The models extensively relied on weekly NDVI, climate, DEM and weather variables to classify the crop classes. July and August NDVI layers were the most important variables for both PCMod and OCMod. The majority of variables included in this study were utilized over 70% by the models (see under ‘Attribute usage’ in Supplementary Outfile S1 and Supplementary Outfile S2). This attribute usage identifies the importance of the inter- and intra-annual samples of attributes such as NDVI (vegetation conditions), climate and weather, and geographical parameters (DEM, slope) for identifying crop types. However, soil properties (SSURGO) and regional variables (ECO and MLRC) were less utilized by the models than we previously thought. Other potential variables like latitude and recently developed 30 m soil map, POLARIS^45^, could possibly have higher impact on the models. However, excessive input variables will increase the chance of over-fitting so replacing existing inputs with similar or improved inputs could be a way forward for future model enhancement efforts. For example, ECO and MLRC could be replaced by latitude so regional variance along with sun angel and day length seasonality differences could be captured efficiently. Additionally, SSURGO could be replaced by POLARIS or other high resolution digital soil maps.

Accuracy results from our study closely followed a trend that is typically observed in the area of crop cover classification – the major, most abundant crop types, such as corn, soybeans, and wheat are more accurately classified compared to the minor crop types^3,44^. Similarly, Wardlow and Egbert^4^ observed that croplands with smaller patches that tend to have more mixed pixels, leads to modelling confusion and lower mapping accuracy, while on the other hand, large contiguous areas of crops tend to carry higher mapping accuracies. Wardlow and Egbert^4^ also pointed out that fallow and unplanted fields (hay) have highly variable multi-temporal NDVI, which can confuse crop classification algorithms.

**Figure 7:**
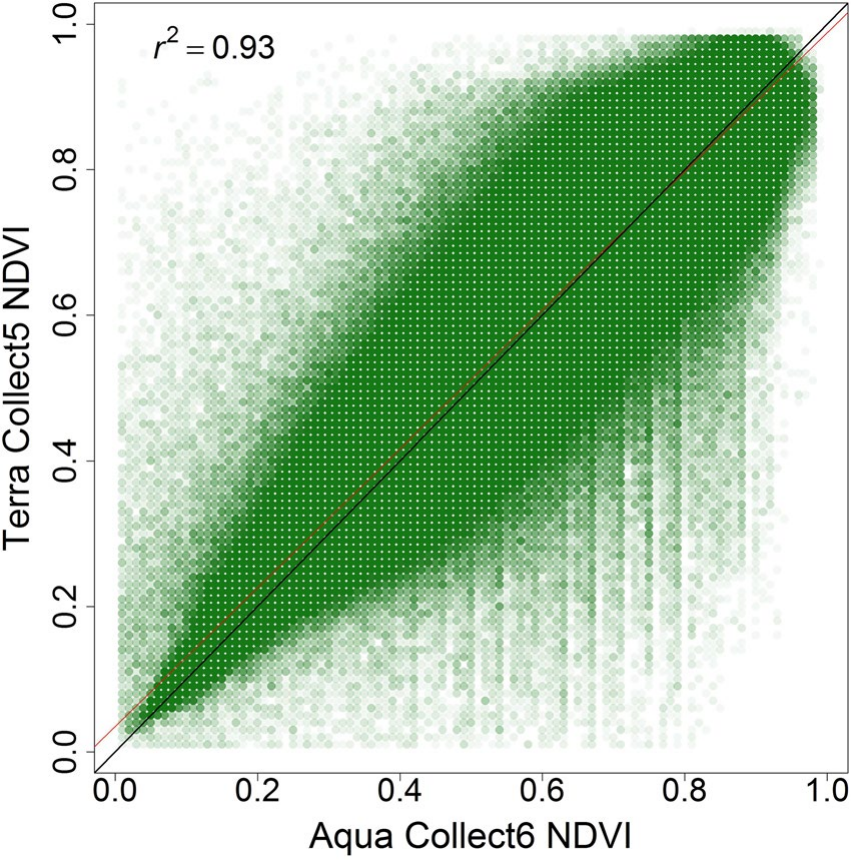
Comparison of CONUS NDVI of eMODIS Terra Collect 5 (y-axis) and eMODIS Aqua Collect 6 (x-axis) for 2014 growing season. Black line is 1:l and red line shows regression fit.

We observed unexpected lower accuracy in 2009 and a downward trend after 2012 (Fig. 3). Our model was trained using CDL data from 2008–2013, which may not provide a sufficient range to capture enough weather and phenological variability. We believe our models failed to capture a record anomaly of 2009. The USDA reported that 2009 had an abnormally wet and cool spring, summer, and autumn, causing delay in major crops (corn and soybean) planting, maturation, and harvesting in the cornbelt. Corn and soybean together account for over 30% of the total crop area in CONUS and increases every year. However, these two crops had record breaking high production for the same year^46^. The phenological similarity of some crops (e.g. corn and soybeans; wheat and barley) means subtle phenological changes could lead to completely different results. Merging these problem crops similar to what Massey *et al*.^47^ did in their study, might improve overall mapping results. Zhong *et al*.^48^ found that when one year’s training samples of corn and soybeans were applied to model another year’s corn and soybean within a single county, the overall accuracy went down by an average of 5 points. We also could take into account the changing methodology and production results of both our independent and dependent variables in an attempt to normalize any variations. The NASS CDL (dependent variable), for example, has been updated and improved over time^3,31,42^. Another example is a problem with the eMODIS NDVI (primary independent variable), which was used for developing CCM model. The Terra satellite from which all eMODIS products are derived is drifting and this means the eMODIS products could be slowing or changing/degrading. As a result, the eMODIS Aqua Collect 6 has been suggested as a replacement for 2014 and beyond^49^. Also, processing of MODIS Collect 5 products has been stopped as of March 2017 with intension to decommission all of the products beginning in fall 2017, making Collect 6 products the only option^50^ going forward. Figure 7 illustrate the difference between weekly eMODIS Terra Collect 5 and weekly eMODIS Aqua Collect 6 NDVI. Table 4 shows how those differences affected our mapping accuracy results. The map accuracy of 2014–2016 products using eMODIS Terra Collect 5 were still not as strong as the accuracy of the training years but substantially better than with the Aqua Collect 6. Furthermore, with the improvement of crop genomics and farming techniques, crop traits such as phenology and physiology might change rapidly in the future^51^. These examples clearly illustrate the need to consistently process and normalize all of the input data in order to achieve consistent results. Therefore, we plan to implement the following updates to our methodology moving forward: 1) update training data each year using the CDL layer of the previous year, and 2) recalibrate the model with eMODIS Aqua Collect 6 NDVI after replacing eMODIS Terra Collect 5 NDVI.

**Table 4:** Comparison of overall map accuracy of Terra Collect 5 and Aqua Collect 6 for three years with all other variables except eMODIS NDVI remaining the same for all crop cover classes.

Year	Terra C5	Aqua C6
2014	59.62%	54.9%
2015	57.57%	52.97%
2016	58.03%	57.11%

## Conclusion

This study demonstrated the strong potential of producing rapid crop cover maps for the CONUS. These timely products could facilitate other near real time assessments such as carbon flux, water use, and assessment of policy and economics on farm management. All data sources, including eMODIS NDVI, weather and climate data (PRISM), and elevation, used in this study are publicly available at no cost. Annually, crop cover maps with 250 m spatial resolution could be generated by the beginning of September, before harvesting begins for most crops. While testing the rapid mapping approach, this study produced crop cover maps for 2008–2016, which have moderate overall mapping accuracies^52^; however, the accuracies could be improved by annually updating the sample data, incorporating sample points from the previous more recent years, and redeveloping the CCM model. The current approach included only a handful of crop types; however, additional crop types that are included in NASS CDL could also be included in the CCM and mapped in a similar, rapid manner.

## Electronic supplementary material

Supplementary Figure S1

Supplementary OutFile 1

Supplementary OutFile 2
