# Supplementary material for: Rapid Crop Cover Mapping for the Conterminous United States
Source: Sci Rep. 2018 Jun 5;8:8631. doi: 10.1038/s41598-018-26284-w (PMC5988726; doi:10.1038/s41598-018-26284-w)
Supplement: Supplementary file 1 — Supplementary Figure S1 [file 41598_2018_26284_MOESM1_ESM.pdf]

Rapid Crop Cover Mapping for the Conterminous United States.

Devendra Dahal[a,*], Bruce Wylie[b], and Danny Howard[a].

[a] *Stinger Ghaffarian Technologies (SGT) Inc., Contractor to U.S. Geological Survey (USGS) Earth Resources Observation and Science (EROS) Center, Sioux Falls, SD, USA; devendra.dahal.ctr@usgs.gov (D.D.); danny.howard.ctr@usgs.gov (D.H.)*

[b] *USGS EROS Center, Sioux Falls, SD, USA; wylie@usgs.gov*

* *Correspondence: devendra.dahal.ctr@usgs.gov; Tel.: +1-605-594-2716*

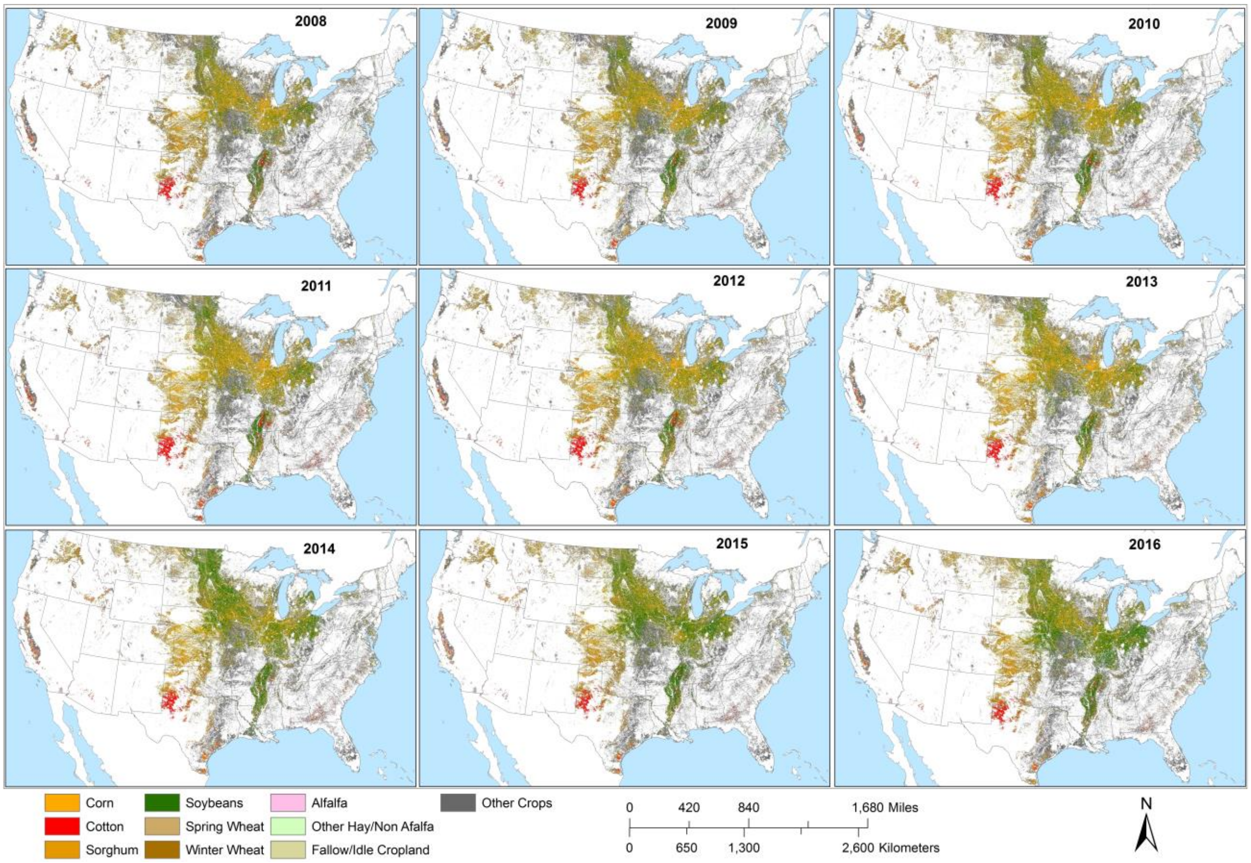

Supplementary Figure S1: Spatial distribution of CONUS rapid crop cover map products of SepNP2m model generated as of the 1st of September (during growing season) of years 2008 – 2016.
